# Anatomical Variations in Morphometric Measurements of the Coracoid Process in a Cross Section of the Sudanese Population: Evaluation Using Chest Computed Tomography

**DOI:** 10.7759/cureus.74085

**Published:** 2024-11-20

**Authors:** Ahmed Abdelrahman, Ali E Mohamed, Khalid Salih, Mahmoud M Abdelrahman, Abubakr Muhammed

**Affiliations:** 1 Human Clinical Anatomy, Faculty of Medicine, National University, Khartoum, SDN; 2 Medicine and Surgery, Faculty of Medicine, University of Khartoum, Khartoum, SDN; 3 Trauma and Orthopedics, United Lincolnshire Hospitals NHS Trust, Grantham, GBR; 4 Trauma and Orthopedics, Faculty of Medicine, Nile University, Khartoum, SDN; 5 Surgery, University of Gezira, Madani, SDN

**Keywords:** anatomy, computed tomography (ct), coracoid process, measurements, morphometric variations, orthopedic surgery, radiology, scapula, sudanese, sudan khartoum

## Abstract

Introduction

The coracoid process is integral to the functionality of the scapula, serving as a crucial attachment point for several muscles involved in shoulder movement and stability. In pathologies and fractures of the coracoid process, understanding the morphometric variations is essential for devising optimal surgical strategies. Given the substantial lack of relevant data, this study aimed to analyze the morphometric variations in the dimensions of the coracoid process among the Sudanese population and evaluate the differences in the measurements in relation to gender.

Methods

The study was performed on 100 images of human scapulae (50 males and 50 females). The radiographs and reports were acquired from the Radiology Department at Almoalim Medical City, Khartoum, Sudan. CT scan images were uploaded to medical imaging software (PaxeraViewer version 1.0.1.9, PaxeraHealth, Newton, MA, USA). Quantitative measurements of linear parameters were calculated via this software, and data was analyzed using SPSS Statistics version 23 (IBM Corp. Released 2015. IBM SPSS Statistics for Windows, Version 23.0. Armonk, NY: IBM Corp.).

Results

Our study revealed that the mean measurements of the coracoid process dimensions were as follows: the length 39 ± 2.7 mm, the tip thickness 10.8 ± 1.8 mm, the base height 13 ± 1.1 mm, and the base width 22.2 ± 1.6 mm. Gender-based comparisons showed a trend towards larger parameters in males compared to females. Significant variations in the length (p = 0.03) and base height (p = 0.002) of the coracoid process were noted.

Conclusion

This study demonstrated variations in coracoid process dimensions among the Sudanese population, emphasizing gender influence. Moreover, comparisons to earlier research highlighted discrepancies across different ethnicities. Further investigation with a greater number of cases from a prospective viewpoint is needed for more compound insight into this issue.

## Introduction

The skeletal shoulder develops through both types of ossification processes. In the clavicle, bone is directly laid down into the mesenchyme in a process known as intramembranous ossification. The remaining shoulder bones are formed by endochondral ossification, which involves the replacement of hyaline cartilage with bone. The glenohumeral joint connective tissues develop from the mesodermal germ layer [1]. The shoulder is a highly mobile joint comprising three main bones: the humerus, scapula, and clavicle, which form the glenohumeral, acromioclavicular, and sternoclavicular joints. The scapula is a large, triangular, flat bone located on the posterior chest wall, corresponding to the second to seventh rib. It has two surfaces, three borders, three angles, and three processes (the spine, the acromion, and the coracoid) [2]. The glenohumeral joint, a ball and socket joint between the humerus and the glenoid cavity of the scapula, allows extensive movement in multiple planes. Stability is provided by the rotator cuff muscles (supraspinatus, infraspinatus, teres minor, and subscapularis), surrounding ligaments, and the labrum, a fibrocartilaginous ring that deepens the socket. Blood supply comes from the subclavian and axillary arteries, while nerves from the brachial plexus enable sensation and motor function. This anatomy allows remarkable flexibility, though it also makes the shoulder prone to injuries [2,3].

Scapular developmental disorders include a variety of diseases, ranging from the common Sprengel deformity (an elevated scapula frequently associated with anomalies of the cervical spine) to less common malformations such as Kosenow syndrome (scapular and pelvic hypoplasia) and scapular duplication. While the majority of abnormalities are functionally insignificant, some can cause disability, especially when paired with additional systemic or skeletal challenges [4]. Other relevant disorders include congenital absence of the scapula, which is frequently linked to the absence of the upper limb, and congenital glenoid dysplasia, which results in shoulder instability due to lack of glenoid epiphysis formation. More common abnormalities, such as nonunion of ossific centers (affecting the glenoid, acromion, or coracoid), may occasionally be confused for fractures but typically don't need to be treated. An abnormal version of the glenoid can cause frequent shoulder dislocations, which need surgical correction [5].

The coracoid process is a thick, curved, bird-beak-like projection that arises superolaterally from the upper border of the head and then bends sharply to project forwards and slightly laterally. It is attached by a broad base to the upper part of the neck of the scapula. The component parts of the process are the base, angle, shaft, and apex, respectively. The coraco-glenoid notch is an indentation located between the coracoid process and the glenoid cavity. As the coracoid process projects laterally, it defines the subcoracoid space beneath it [2,6].

The coracoid process is palpable just below the lateral end of the clavicle (collarbone), known as the “surgeon’s lighthouse” because it serves as a landmark to avoid neurovascular damage. The distance between the coracoid base and the neurovascular structures is like a 90-degree chair [7,8]. Major neurovascular structures enter the upper limb medial to the coracoid process, so surgical approaches to the shoulder region should always take place lateral to the coracoid process [7].

The anatomy of the coracoid process and its related structures in the glenohumeral joint is crucial for accurately interpreting radiological images and aiding in surgical procedures to treat various shoulder pathologies [9]. The coracoid process serves as an attachment point for three significant muscles: the coracobrachialis, which aids in arm flexion and adduction at the shoulder; the short head of the biceps brachii, involved in elbow flexion and forearm supination; and the pectoralis minor, which stabilizes the scapula by drawing it anteriorly and inferiorly. Understanding these muscular attachments, their functions, and their development is essential for diagnosing and managing shoulder conditions effectively. For instance, potential differences in the pectoralis minor muscle that result from developmental defects may sometimes present as asymptomatic conditions or predispose people to functional impairments, shoulder-related disorders, rotator cuff dysfunction, and shoulder impingement [8,10]. Additionally, the coracoid is an essential anchor for many ligamentous and tendinous attachments. These consist of the coracoclavicular, coracohumeral, coracoacromial, and transverse scapular ligaments, as well as the tendons of the pectoralis minor, coracobrachialis, and short head of the biceps brachii muscles [8]. The study of the dimensions of the coracoid process can assist orthopedic surgeons in drill-hole placement, prosthetic fixation, and the Latarjet-Bristow operation with the prediction of the safety margin for coracoid transfer [11-14]. Morphology of the coracoid process plays an important role in understanding impingement syndrome, the pathogenesis of rotator cuff diseases, and multiple ligamentous conditions [15-18]. This study aimed to analyze the morphometric variations in the dimensions of the coracoid process among the Sudanese population and evaluate the differences in the measurements in relation to gender.

## Materials and methods

This study was a retrospective, analytical, facility-based, cross-sectional study conducted in Sudan's capital, Khartoum. It included 100 CT chest images from patients without a history of shoulder trauma, pathology, or previous scapular surgery who underwent CT scans at Almoalim Medical City in 2022, and the measurements were conducted on the right side. Ethical approval was obtained from the Almoalim Medical City Administration and the Federal Ministry of Health, Khartoum, Sudan (IRB No. KHREC-0163-2023). In compliance with the Declaration of Helsinki, participants were fully informed, provided consent, and assured confidentiality and the right to withdraw. Data security and anonymity were strictly maintained to uphold ethical standards.

**Figure 1:**
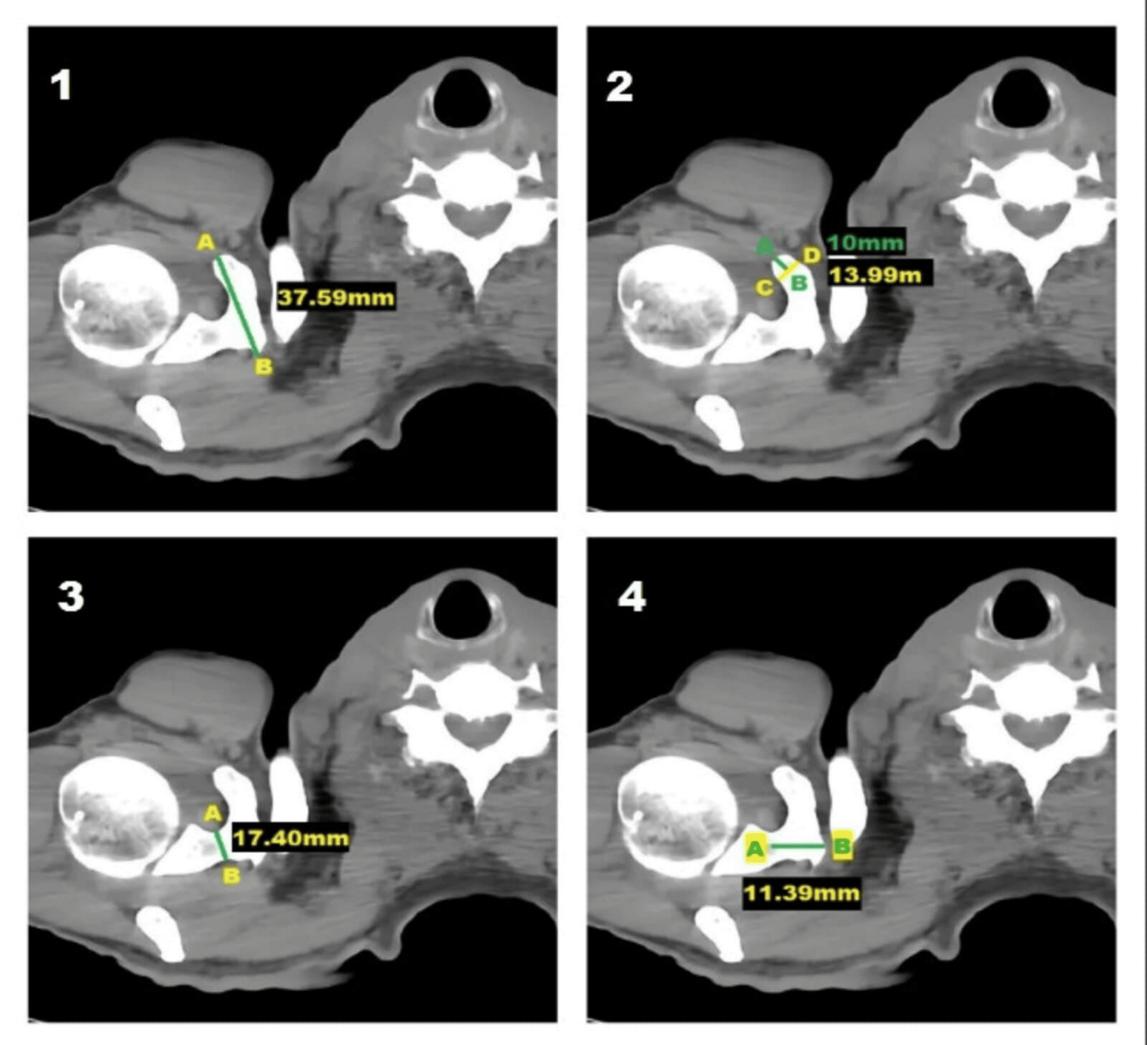
Axial CT scan image showing the measurements of the right coracoid process: (1) length, (2) tip thickness, (3) base height, and (4) base width CT axial section at the level of T1 CT: computed tomography

The coracoid process and relevant anatomical landmarks on the scapula were identified, and these anatomical landmarks were used across all the measurements by a single investigator to ensure the accuracy of the measurements recorded (Figure 1).

CT scan images were uploaded in DICOM format in PaxeraViewer version 1.0.1.9 (PaxeraHealth, Newton, MA, USA); consequently, quantitative measurements of linear parameters were calculated. PaxeraViewer is an application used for viewing and manipulating medical images like CT, X-ray, and US. Users can perform adjustments, measure regions of interest, and make various image alterations.

Parameters measured (in millimeters) were the length of the coracoid process (distance from the tip to the end of the horizontal part), tip thickness of the coracoid process (superoinferior distance 1 cm posterior to the tip), base height (maximum superoinferior distance of the base), and base width (maximum anteroposterior distance of the base).

The data were entered into an Excel spreadsheet (Microsoft Corporation, Redmond, WA, USA) and imported for analysis through SPSS Statistics version 23 (IBM Corp. Released 2015. IBM SPSS Statistics for Windows, Version 23.0. Armonk, NY: IBM Corp.). The normality of data distribution was checked by skewness and kurtosis level. The results were presented as mean ± standard deviation (SD), as well as maximum and minimum values. Then, gender differences were evaluated using an independent sample t-test. The results are considered statistically significant when the p-value is less than 0.05 at a confidence interval of 95%.

## Results

Length of the coracoid process

**Figure 2:**
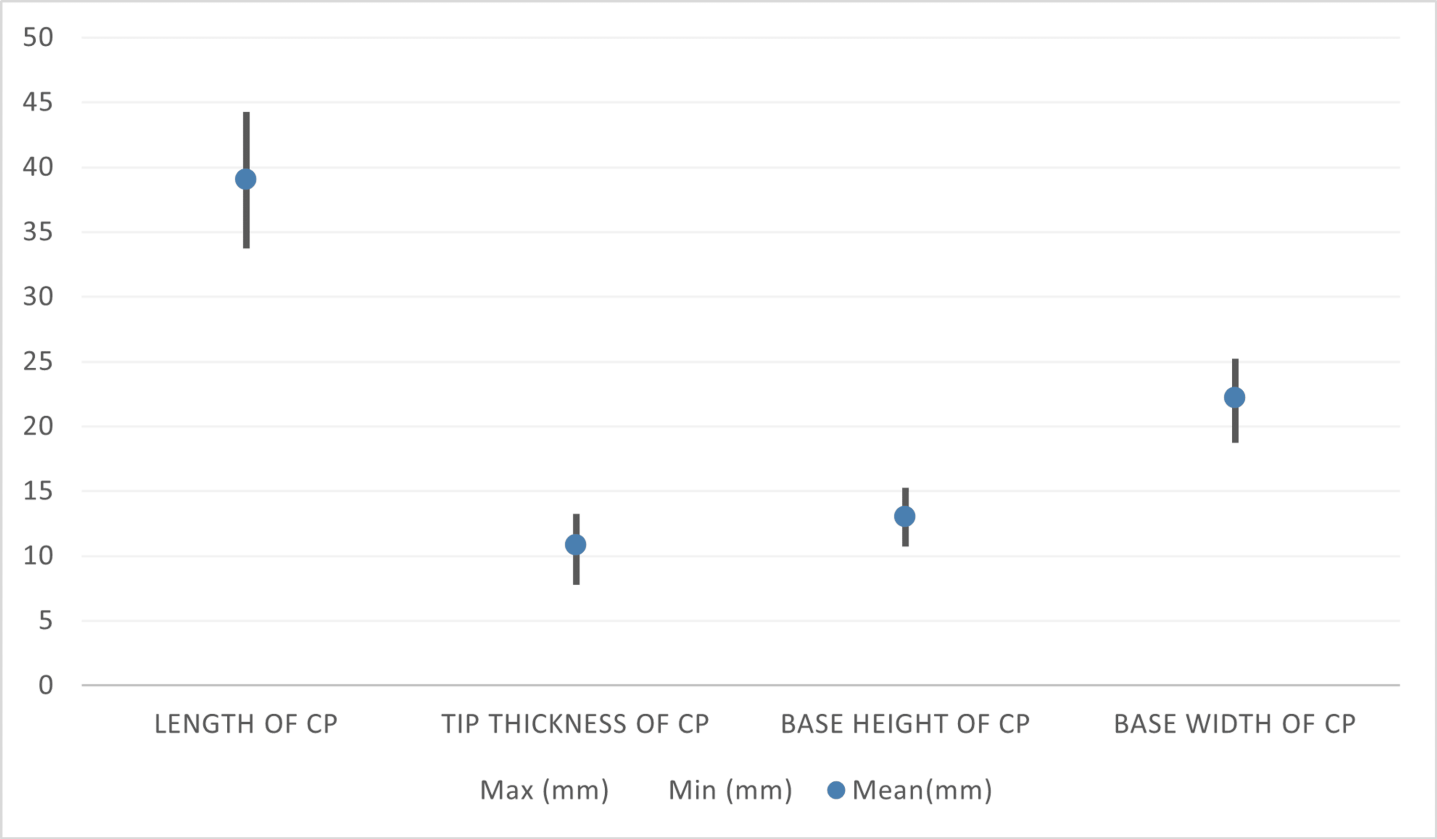
Descriptive statistics of anatomical variation in morphometry of the coracoid process among the Sudanese population CP: coracoid process, Min: minimum, Max: maximum

Out of the 100 coracoid processes measured, the smallest recorded measurement was 34 mm, while the largest was 44 mm. The mean was calculated to be 39 ± 2.7 mm (Figure 2).

Thickness of the tip of the coracoid process

Regarding the tip thickness of the coracoid process, the minimum reading was found to be 8 mm, while the maximum reading was found to be 13 mm. The mean was determined to be 10.8 ± 1.8 mm (Figure 2).

Base height of the coracoid process

The study showed that the biggest measurement was found to be 15 mm, whereas the smallest measurement was found to be 11 mm. The mean base height was 13 ± 1.1 mm (Figure 2).

Base width of the coracoid process

The maximum recorded measurement of the base width was 25 mm in comparison with the minimum recorded measurement of 19 mm. Moreover, the mean was worked out to be 22.2 ± 1.6 mm (Figure 2).

Gender differences in morphometric measurements of the coracoid process

The length of the coracoid process in males was 40.6 ± 2.4 mm in comparison to the females 37.4 ± 1.8 mm (p = 0.03). Additionally, the tip thickness in males was measured to be 11.5 ± 1.6 mm as opposed to the females' 10 ± 1.5 mm (p = 0.645). Moreover, the mean base height in males was recorded as 13.4 ± 1.2 mm in contrast with the females' 12.5 ± 0.9 mm (p = 0.002). However, the analysis furthermore indicated that the mean base width in males was 22.9 ± 1.5 mm compared to that of females 21.6 ± 1.5 mm (p = 0.864) (Tables 1-2). An independent t-test was used to calculate the p-values.

**Table 1:** Descriptive statistics of anatomical variation in morphometry of the coracoid process among males and females CP: coracoid process

Measurements	Number of samples	Minimum (mm)	Maximum (mm)	Mean (mm)	Standard deviation (mm)
Length of CP males	50	37.0	44.0	40.6	2.4
Tip thickness of CP males	50	8.0	13.0	11.5	1.6
Base height of CP males	50	11.0	15.0	13.4	1.2
Base width of CP males	50	20.0	25.0	22.9	1.5
Length of CP females	50	34.0	41.0	37.4	1.8
Tip thickness of CP females	50	8.0	13.0	10.0	1.5
Base height of CP females	50	11.0	14.0	12.5	0.9
Base width of CP females	50	19.0	24.0	21.6	1.5

**Table 2:** Mean, standard deviation, and p-value of the coracoid process among male and female subjects. p-value was calculated using independent t-test CP: coracoid process

Measurement	Male: mean ± SD (mm)	Female: mean ± SD (mm)	P-value
Length of CP	40.6 ± 2.4	37.4 ± 1.8	0.03
Tip thickness of CP	11.5 ± 1.6	10 ± 1.5	0.645
Base height of CP	13 ± 1	12 ± 0.8	0.002
Base width of CP	22.9 ± 1.5	21.6±1.5	0.864

## Discussion

Knowledge of the morphometric dimensions of the coracoid process is pivotal in evaluating pathologies and surgical approaches to the shoulder. Previous research showed variations in these measurements worldwide. This study evaluated these readings in the Sudanese population.

Length of the coracoid process

Our study found that the mean length of the coracoid process in males (40.6 ± 2.4 mm) was significantly greater than that in females (37.3 ± 1.8 mm) (p = 0.03). This was also the case in several previous studies, with notable variability in values across different populations. Firstly, in India, it was reported that the mean lengths were 37.21 ± 3.93 mm and 36.86 ± 4.10 mm for males (right and left sides) and that for females (right and left sides), they were 34.06 ± 3.76 mm and 32.56 ± 2.89 mm [19], which are lower than those obtained in this paper. Furthermore, a Turkish study reported an even shorter mean length of 19.4 mm [20], and another in Thailand reported similarly lower values [21]. Conversely, a study from the USA reported mean lengths of 45.7 ± 3.7 mm for males and 41.5 ± 2.3 mm for females [22], which are quite higher than our findings. Finally, a study in Malaysia found a mean length of 37.94 ± 4.30 mm [23], and another in multiple Asian ethnicities reported a range from 39.19 mm to 43.32 mm [24], which are within our ranges but lacking gender specificity.

Tip thickness of the coracoid process

The mean tip thickness was found to be higher in males (11.5 ± 1.6 mm) compared to females (10 ± 1.5 mm) (p = 645), which is again consistent with previous research. An Indian study found mean tip thicknesses of 8.20 ± 1.20 mm and 8.23 ± 1.06 mm for males (right and left sides) and mean thicknesses of 7.64 ± 0.94 mm and 7.32 ± 0.81 mm for females (right and left sides) [19], which are significantly lower than our values. Another paper in Canada reported a mean tip thickness of 10.5 ± 1.7 mm [25], which is midway between our male and female calculations.

Base height of the coracoid process

In our study, the mean base height was significantly bigger in males (13 ± 1 mm) than in females (12 ± 0.8 mm) (p = 0.002). This finding is close to that of a study in Germany, which found mean base heights of 15.4 ± 1.3 mm for males and 13.6 ± 1.7 mm for females [26]. In contrast, an Indian study reported mean base heights of 20.62 ± 2.57 mm and 20.59 ± 3.63 mm for males (right and left sides), and 19.91 ± 3.08 mm and 18.78 ± 2.50 mm for females (right and left sides) [19], which is significantly higher than our findings.

Base width of the coracoid process

The mean base width in our study was larger in males (22.9 ± 1.5 mm) than in females (21.6 ± 1.5 mm) (p = 864). This was the case in India, where a study showed 15.28 ± 1.70 mm and 15.13 ± 1.73 mm for males (right and left), and 13.51 ± 1.75 mm and 13.08 ± 1.07 mm for female counterparts (right and left) [19]. A study carried out in Germany also supported our deductions, revealing that male bases measured 16.7 ± 2.9 mm and that female bases were 13 ± 1.7 mm [26]. Both of these studies also showed a significant reduction in values compared to our own.

Our results are consistent with the overall pattern noted in earlier research, suggesting that male coracoid processes are often larger than those of females [27].

The outbreak of war in Sudan led to the destruction of facilities and servers at Almoalim Medical City, preventing the collection of additional data. This led to a relatively small sample size of 100 CT scans that might not adequately represent the broader Sudanese population, potentially limiting the generalizability of the results. A larger sample size would have enhanced the reliability and validity of the conclusions derived from this study. Additionally, stratifying the results according to age group ranges and tribal associations would have more properly reflected the results; however, this information was not available in the institution's data.

## Conclusions

This study offers important information about the morphometric differences of the coracoid process among the Sudanese population by demonstrating statistically significant differences in length and base height between genders. These results are consistent with patterns seen in other previous research. To improve surgical techniques for shoulder joint treatments such as the Latarjet-Bristow operation and coracoid transfer, it is crucial to gain a deep understanding of these discrepancies. The information also emphasizes how essential it is to take demographics into account while organizing surgical procedures in order to guarantee the best possible results. Our research contributes to the expanding collection of information on shoulder anatomy, paving the way for more effective and accurate surgical interventions.
